# The effect of healthier menu item price reductions in the out-of-home food sector on energy purchased and consumed: a restaurant-based pilot experiment

**DOI:** 10.1186/s12889-025-21992-1

**Published:** 2025-05-22

**Authors:** Rozemarijn Witkam, Jane Brealey, Rebecca Latham, Andrew Jones, Eric Robinson

**Affiliations:** 1 Department of Psychology, Institute of Population Health, University of Liverpool, Liverpool, UK; 2 School of Psychology, Faculty of Health, Liverpool John Moores University, Liverpool, UK

**Keywords:** Nutrition intervention, Fiscal policy, Obesity, Out-of-home food sector

## Abstract

**Background:**

Frequent out-of-home food sector (OOHFS) use is associated with poor dietary intake and obesity. There are limited real-world studies on pricing interventions to encourage healthy eating in the OOHFS. We performed a pilot study to collect preliminary trial data on the potential impact of a price reduction intervention on healthier menu items on purchasing and consumption of kilocalories (kcal) in a full-serviced restaurant among people from both lower and higher socioeconomic position (SEP).

**Methods:**

The main trial design was a pre vs. post price reduction comparison (within-subjects), where participants (adults aged ≥ 18 years) received a control menu with standard pricing at visit 1 and a menu with price manipulations (30% reduction for healthy items) at visit 2. A sub-study was conducted with a comparison sample to estimate potential pre-post changes to outcome variables in the absence of a pricing intervention. Linear mixed models assessed pre-post changes in kcal purchased and kcal consumed.

**Results:**

In total, 114 participants were recruited; 92 were randomised to the main study and 22 to the comparison sub-study. Of those participating in the main study, 78 completed and 14 were lost to follow-up. There were no participants lost to follow-up in the comparison study. Of the completers, 46 participants were considered higher SEP (i.e. bachelor’s degree or higher) and 32 participants were lower SEP (i.e. some college or associate degree or lower). Kcal purchased and consumed decreased from visit 1 to visit 2; however, this reduction was only statistically significant for total kcal consumed (regression coefficient: -98.0 (95% CI -181.9, -14.2), *p* = 0.02). There were no notable intervention effect differences between higher and lower SEP participants, but we were under-powered to formally test for SEP differences. In the comparison study, kcal purchased and consumed was also lower at visit 2 compared to visit 1, although this should be interpreted with caution due to the small sample size.

**Conclusion:**

A price reduction intervention on lower energy menu items is potentially effective in encouraging healthier eating in a restaurant setting. Larger studies with inclusion of a control group (e.g., randomised controlled trials) are now needed to confirm intervention effects and whether they are equitable across different socioeconomic groups.

**Supplementary Information:**

The online version contains supplementary material available at 10.1186/s12889-025-21992-1.

## Introduction

Poor diet (e.g., energy-dense, low in fibre, high in saturated fat and high in salt) is the second and the third largest risk factor for deaths worldwide for women and men respectively according to The Global Burden of Disease collaboration 2019 [1]. Diet is also a major cause of non-communicable diseases, such as obesity, cardiovascular disease, diabetes and cancer [2]. Total annual healthcare and societal costs of obesity alone in the UK has been estimated to be around £58 billion [3]. Those with lower socioeconomic position (SEP) are more likely to have poorer diets and obesity, contributing to socioeconomic disparities in health [4–11].

The out-of-home food sector (OOHFS) has an important impact on national diet and is now considered to be a policy context in which overweight and obesity should be addressed [12]. More than a quarter (27%) of the UK adult population eat in the OOHFS – such as restaurants, cafes and food delivery services – at least once per week [13]. Foods supplied in the OOHFS are often high in energy [14, 15] and eating out of the home has been linked to excessive energy consumption [16] and obesity [17]. There are currently limited public health policies which address the OOHFS. Mandatory calorie labelling was recently introduced in the OOHFS in the UK as an intervention to encourage healthier eating [18, 19]; however, the effect of calorie labelling on diet has been shown to be relatively small [20–25]. Moreover, information-based interventions like calorie labelling could theoretically widen dietary inequalities because when making food choices, people from lower SEP are less motivated by health and weight control [26] and more motivated by price [27] compared to those from higher SEP.

An alternative approach to information-based interventions is the use of fiscal strategies to improve diet [28–30]. Fiscal strategies can include price increases to unhealthy food (e.g., taxation) or price reductions to healthier food (e.g., subsidies) to encourage consumers to improve dietary choices. A 2017 review found that price decreases may be more effective than price increases in encouraging healthier diets [31] and given concerns over the financially regressive nature of taxation of less healthy food on lower income groups [32], this suggests that reducing the price of healthier food options may be a promising public health approach.

A recent systematic review and meta-analysis (*n* = 14) concluded that price reduction interventions may be equally effective across different socioeconomic groups [28]. A standardised 20% price reduction led to an increase of fruit and vegetable purchases of 17.3% in the general population and had a similar effect in low-income populations (15.6%) [28]. However, it has been proposed that price reductions to healthier foods may have more benefit in lower income groups due to existing inequalities in diet quality [28, 33, 34]. Most price reduction studies to date have focussed solely on fruit and vegetables purchases and were implemented in supermarkets or (workplace) canteens [28]. There is a lack of evidence on whether price reduction interventions are also effective and equitable in the OOHFS and the extent to which they can alter energy intake. Furthermore, a relatively large number of studies on dietary interventions in the OOHFS, including pricing studies, use hypothetical or laboratory-based methodologies [33, 35–37] which have limited ecological validity. Therefore, there is a need for real-world studies.

As foods in the OOHFS are known to be high in energy [14, 15] and contribute to excessive energy intake [16], we designed a price reduction intervention based on menu item energy content and examined its potential impact on energy purchased and consumed. To further understand implications of the intervention on general health and obesity prevention, we also studied the potential effect of the intervention on total sugar, fat, saturated fat and salt intake and compensatory energy intake later in the day.

We originally planned a large pre-post trial to understand the effect of a restaurant price-reduction intervention on healthier eating. However, due to unforeseen financial circumstances, the restaurant was forced to close during data collection. In the present research we therefore treated data collected as a pilot. Consistent with guidelines on implementation pilot studies of interventions [38], the aims of the present study were 1) to collect preliminary trial data examining the potential impact of a price reduction intervention on purchasing and consumption of energy in the OOHFS among people from both lower and higher SEP groups; and 2) to collect sufficient data to be able to inform effect size estimation for a future definitive trial, if appropriate.

## Methods

### Setting

This study was run in an independent, medium-sized, full-service restaurant in the city centre of Liverpool, UK. The restaurant had a varied menu but consisted mainly of typical UK casual-restaurant food, including sandwiches, fish and chips, pastas and salads (see supplementary materials, Figures S1 and S2 for menus).

### Design

We originally designed a trial to compare the effect of reducing the price of healthier menu items on a restaurant menu (pre-post price reduction: within-subject factor) and stratified recruitment by SEP (high vs. low SEP: between-subject factor). See https://osf.io/7vh3p/ and https://clinicaltrials.gov/study/NCT05818345 for full details. However, the collaborating restaurant was forced to close during data collection due to unforeseen financial circumstances and this meant that it was not possible to recruit the number of participants required for the full trial. We therefore adapted the study to be consistent with a pilot study. This pilot study is therefore seen as preparatory and exploratory work for a larger trial and is reported according to the Consolidated Standards of Reporting Trials (CONSORT) extension to pilot and feasibility trials [39].

The design of the pilot study retained the pre-post price reduction comparison (within-subject effect) to examine the effect of price reduction intervention on energy purchased and consumed. However, as the closure of the restaurant limited sample size recruited, we were unable to statistically examine whether the effect of intervention significantly differed by SEP (within-between interaction) and in the present pilot study we report data on outcomes by SEP for descriptive purposes.

The trial design was not a randomised controlled trial (RCT) due to constraints on resources. To in part address this limitation, we recruited and randomised participants to a smaller sub-study group (comparison sample) to examine pre-post changes to outcome variables in the absence of a pricing intervention (i.e. pricing remained constant across study visits). Approximately 20% of participants were randomised to this comparison group and we planned to examine results of this comparison group separately.

See schematic overview of the study design in the Supplementary Materials, Figure S1.

### Recruitment and participants

We recruited participants from the local community via social media adverts on Facebook and Instagram between March–June 2023. We also contacted people from an existing database of individuals who participated in previous studies and registered that they were interested in participating in future research. Participants were eligible if they were a UK resident, aged 18 years and older, fluent in English, have an out-of-home meal at least once a month and had no dietary allergies. There were no exclusion criteria based on dietary patterns, such as vegetarianism. Participants were instructed to visit the restaurant two times, 1–6 weeks apart, where they would purchase and consume a meal and answer questions on their demographic background and eating habits. They were allowed to bring a maximum of three other guests with them to the restaurant, who were also recruited to the study. Participants were aware that they would be reimbursed £20 per restaurant visit when they signed up (see Procedures section for detailed information).

Recruitment was stratified by SEP using education level (50:50, high vs. low SEP). Education was considered a suitable stratification measure because of the clear relationship between educational attainment and poor diet [40] and obesity [4]. Education level also correlates with other SEP indicators, such as income and occupation [41]. Consistent with previous studies [35, 37], a higher level of education was defined as a bachelor’s degree or higher and a lower level of education was defined as some college or associate degree (equivalent to A-levels) or lower. We limited the number of university students eligible to participate to approximately 10% to ensure sample size was not predominantly drawn from students, as opposed to the general public.

Participants were randomised by the researchers at the level of participant group (i.e., per table) 4:1 into either the intervention study or the comparison sub-study using the RANDBETWEEN function in Excel and were stratified 1:1 by low vs. high SEP. Participants were not aware whether they were participating in the intervention study or the comparison sub-study.

### Intervention

The intervention was a 30% price reduction on healthier menu items based on energy content. Previous studies implemented price reductions ranging from 10–50% [42–48] and we adopted a 30% reduction to be broadly comparable with these studies. Main meal menu items of 600 kcal or less were discounted, based on the recommendation from Public Health England that lunch or an evening meal should consist of no more than 600 kcal [49]. Side dishes were discounted if they contained 200 kcal or less, which gave an equal split between healthier vs. less healthy side dishes on the menu. Desserts were not discounted as there were no low kcal options (i.e. 200 kcal or less). A standard drinks menu was provided at each visit.

Because participants visited the restaurant twice between 1–6 weeks apart and we reasoned that ordering from the same menu with vs. without price reductions may increase the likelihood of study hypotheses being transparent, we used a counterbalanced design for the food menus that participants ordered from. Two different menus were created (menu A and menu B), with an equal amount of higher and lower kcal dishes: 11 sandwiches/mains (of which 5 lower kcal) and 6 sides (of which 3 lower kcal). The desserts were the same on each menu. The order of menus (AB vs BA) was randomised by participant group, resulting in 50% of participants receiving the pricing intervention (second visit) for menu A and menu B.

The full menus used are in the supplementary materials (Figure S2 and S3).

### Procedures

A link to a short online pre-screening questionnaire was included on online and physical advertisements, which included questions on the eligibility criteria (UK resident, age and dietary restrictions) and education. If potential participants were eligible and willing to participate, they were invited to a full-service restaurant (in Liverpool, UK) twice. Visit 2 was a minimum of one and a maximum of six weeks later on the same weekday and at the same time as visit 1.

#### Visit 1 (control)

On arrival at the restaurant for visit 1, participants were given information about the study and gave verbal consent.

Participants were asked to order lunch (between 12 and 3 pm) or dinner (between 5 and 7 pm) from handheld menus provided on tables. Participants ordered in groups at individual tables and there were no limits on the amount of food and drink ordered by individual participants or groups. A member of the research team took orders and restaurant staff prepared all food and drinks. Prior to food and drinks being served and after tables had been cleared by restaurant staff, a member of the research team photographed ordered items.

After participants finished eating, they completed a baseline questionnaire on demographic characteristics using a tablet device. The researcher then verbally asked whether any food and drinks were shared between participants; and if so, which items and how much. The next morning, participants received a link for the dietary recall questionnaire (Intake24 [50]) and were asked to complete it (for any food and drink consumed after the restaurant visit) by the end of the day.

Participants were reimbursed £20 for participation and this amount was removed from individual customer bills. If participants spent more than £20, they paid the remainder of the bill and if less then £20 was spent, participants were reimbursed the remaining funds.

#### Visit 2 (pricing intervention)

At visit 2, the procedure was identical to visit 1. However, no baseline questionnaire was completed and the following day, after completion of the dietary recall questionnaire, participants reported what they believed the aim of the study was and were then debriefed of the study aims.

### Variables

#### Primary outcome measures, measured at visit 1 and 2

Primary outcome measures were total kcal purchased and total kcal consumed per participant, measured at both visit 1 and 2. Total kcal purchased was determined based on all menu items ordered, including mains, sides, desserts and drinks. Energy content of menu items was obtained through laboratory bomb calorimetry. Energy content of drinks were based on product data on manufacturer websites if branded and estimated using Nutritics [51] for smoothies and hot drinks (ingredients were provided by the restaurant).

Total kcal consumed was derived from the order and an estimation of how much of the meal was consumed. Researchers photographed meals before and after consumption, in addition to asking participants at the end of the study whether and what proportion of dishes were shared on tables. A researcher estimated the percentage of dishes consumed using the above information and 10% was cross-checked by a second researcher, to confirm accuracy. If it was an integrated dish (e.g. pasta or risotto), the percentage consumed was applied to the total kcal of the dish to calculate the kcal consumed. If it was a composite dish (i.e. made out of separate elements, such as a burger with chips or fish and chips), the percentage consumed of each element was estimated separately.

#### Secondary outcome measures, measured at visit 1 and 2

Secondary outcomes included total sugar, fat, saturated fat and salt consumed; later intake in kcal after the restaurant visit; and total money spent.

Total sugar, fat, saturated fat and salt of the dishes were estimated using Nutritics [51], using the ingredient list provided by the restaurant. Consumption was then estimated using photographs (see section primary outcome variables for full detail).

Later kcal intake for the same day following the restaurant visit was measured using a validated dietary recall questionnaire (Intake24, https://intake24.co.uk/) [50], which participants completed the following day for the time period between the visit and the end of each study day.

Total money spent (in £) at each visit was determined based on the total food and drink order of the participant.

#### Participant characteristics and socioeconomic variables, measured at visit 1

In the baseline questionnaire at visit 1, participants were asked to report their age (continuous, years), sex (categorical – male, female), ethnic group (categorical – White; Black/African/Caribbean/Black British; Asian/Asian British; Mixed/Multiple ethnic groups, Other), height (continuous, meters) and weight (continuous, kg). Using self-reported height and weight, body mass index (BMI) was calculated. Weight categories were defined according to WHO cut-off points: underweight (BMI < 18.5 kg/m^2^), normal weight (BMI 18.5–24.9 kg/m^2^), overweight (BMI 25.0–29.9 kg/m^2^) and obesity (BMI ≥ 30.0 kg/m^2^).

Participants were further asked what their highest educational qualification is, with six answer categories: less than high school (1); high school completion (2); some college or associate degree (equivalent to A-levels) (3); bachelor’s degree (4); master’s degree (5); doctoral degree (6).

Employment status categories included full-time; part-time; student; retired; temporary / permanently sick or disabled; looking after home / family; other.

Equivalised net monthly household income was estimated by dividing the midpoint of monthly household income after tax (rounded to the nearest £100) (categorical—Under £800; £800–£1500; £1600–£2300; £2400–£3100; £3200–£3900; £4000–£4700; £4800–£5500; £5600–£6300; £6400–£7100; £7200 or more, Prefer not to answer) by the weight of all members of the household as recommended by Kuhn [52]. The first adult received a weight of 1.0, the second adult and any persons aged 14 years and older a weight of 0.5, and children younger than 14 years a weight of 0.3.

Area-level deprivation was determined based on the Index of Multiple Deprivation (IMD) (2019) fifths, using postcodes of participants [53]. It measures relative deprivation of small areas in England based on seven domains (income; employment; education; skills and training; health deprivation and disability; crime; barriers to housing and services; and living environment) [53].

The MacArthur Scale of subjective social status was used to measure participants’ perception of their socioeconomic status (based on money, education and jobs) compared to others in society) [54]. Participants were asked to place themselves on a ladder (1–10; 1 = lowest and 10 = highest subjective social status).

#### Other variables

We determined whether participants guessed the aim of the study by asking the open question “what do you think the aim of this study is?” in an online questionnaire after completing the dietary recall questionnaire for study day 2. If participants were able to link pricing of foods with dietary consumption, the participants were considered to have guessed the aim of the study.

### Analysis

The analysis plan for the original trial was pre-registered (https://osf.io/7vh3p/) and deviations made for the purpose of conversion to a pilot study are reported in online supplementary materials, Box S1.

Descriptive statistics (means and standard deviations, frequency counts where appropriate) were used to examine participant characteristics, nutritional intake and money spent at visits 1 and 2, both in the total sample and by SEP.

Linear mixed models were used to assess the effect of the pricing intervention (reference group: control menu at visit 1) on the primary outcomes (total kcal purchased and total kcal consumed) and the secondary outcomes (total sugar consumed, total saturated fat consumed, total fat consumed, total salt consumed, later kcal intake and total money spent). Linear mixed models incorporate both fixed effects (i.e. the intervention) and random effects (i.e. subjects).

There were 14 participants lost to follow-up after visit 1 and consequently had missing outcome variables at visit 2. As these were assumed to be missing at random, values were imputed using multiple imputations using chained equations with 10 datasets [55]. Values of outcome variables at visit 2 were predicted based on age, sex, BMI, educational status and that specific outcome variable at visit 1 (e.g., kcal purchased at visit 2 was predicted based on kcal purchased at visit 1). For the primary outcome variables, the significance level was set at *p* < 0.05 and for the secondary outcome variables, this was set at *p* < 0.01 to account for multiple testing. To ensure robustness of the findings, sensitivity analyses were performed on completers only (no imputation of data) and with participants who correctly identified the study aims excluded. All analyses were performed using Stata v18.

As participants registering interest in the study could bring up to three additional participants (typically friends and/or family), all randomisation occurred at the level of participant group. We therefore tested whether there was a nesting effect for each outcome variable (i.e. whether any variance in the outcome was explained by participant group) [56]. Using the loglikelihood ratio test, we tested the difference between the within-peer group variability and the between-peer group variability, i.e. comparing null models to participants within tables. If statistically significant (*p* < 0.05), this is convincing evidence of a nesting effect. As we did not find any evidence of a nesting effect on any of the outcome variables (total kcal purchased *p* = 0.37; total kcal consumed *p* = 0.43; total fat consumed *p* = 0.23; total saturated fat consumed *p* = 0.13; total salt consumed *p* = 0.72; total sugar consumed *p* = 0.57; kcal intake after visit *p* = 0.24; and total money spent *p* = 0.85), final models were not adjusted for nesting.

### Power

A total of 92 participants were randomised to the intervention study. Based on two pricing studies in the OOHFS [57, 58], we used a medium sized statistical effect (Cohen’s f = 0.22) in our sample size analysis for the effect of price intervention. Using an ANOVA with repeated measures in G*power, we estimated that a minimum sample of 44 would be required for the main effect (within-subjects effect) of the pricing intervention (with a power of 80%, alpha at 5%, with two groups (i.e. high SEP and low SEP) and two measurements (Visit 1 and Visit 2), and correlations among repeated measures was set at 0.5). Therefore, our final sample of 92 participants produced sufficient statistical power for a medium effect of the price reduction intervention in the total study population. We were not powered to detect interaction effects by SEP (see https://osf.io/7vh3p/ for more detail).

## Results

### Baseline characteristics

In total, 244 people completed the online pre-screening questionnaire. Of these, 56 people were available on study dates and agreed to participate in the study. They brought 58 eligible guests with them to the restaurant, resulting in 114 participants randomised at the participant group level into either the main study or the comparison study. From participants randomised to the main study (*n* = 92), 78 completed the study (distributed over 37 participant groups) and 14 were lost to follow-up (distributed over 7 groups). On average, there were 2 participants per group and sitting on each table. *N* = 22 participants (distributed over 10 groups) were randomised to the comparison sub-study. Visits 1 were planned between 29/03/2023–01/06/2023 and visits 2 between 05/04/2023–10/06/2023.

**Fig. 1:**
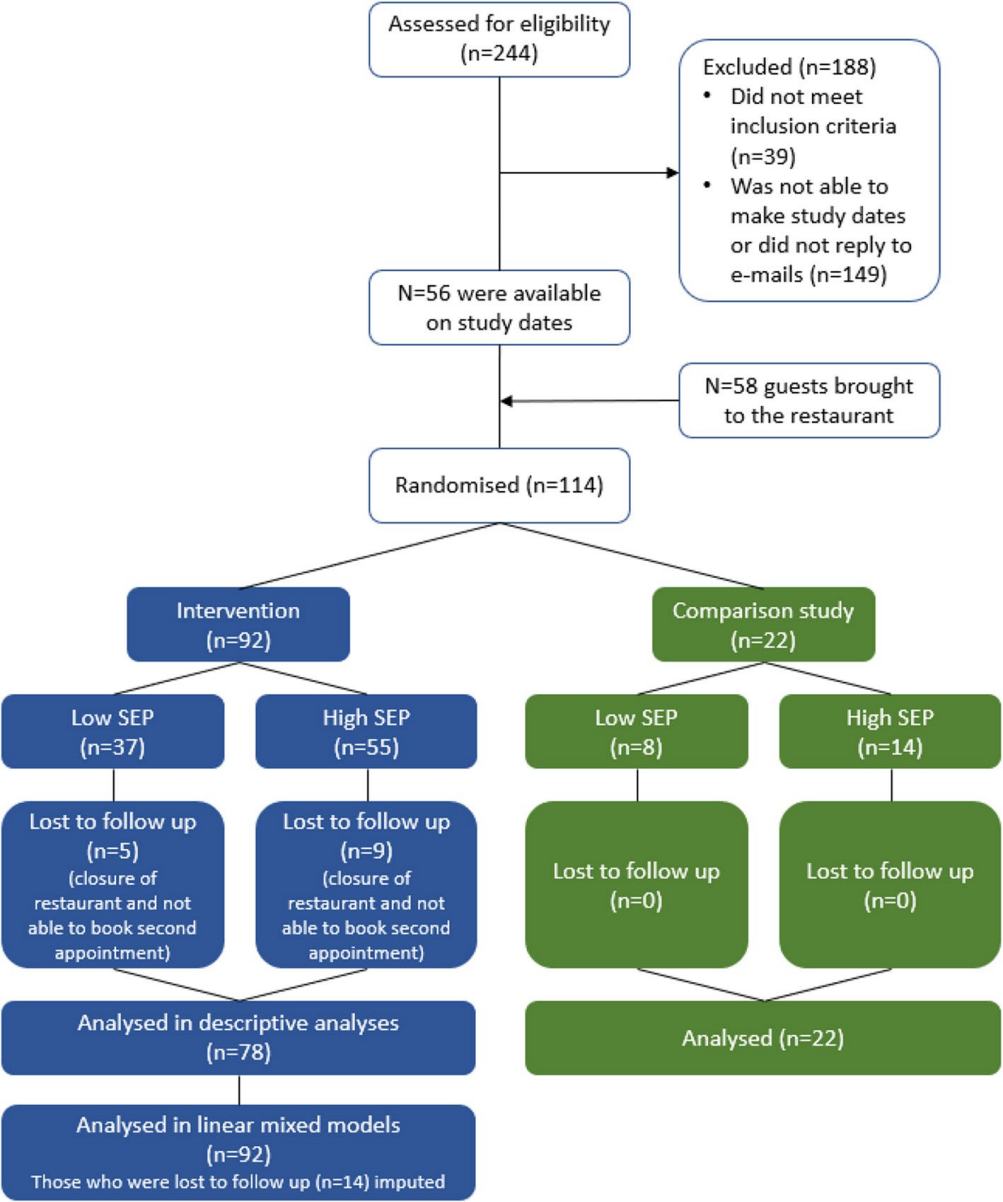
CONSORT flow diagram of study participation

See Fig. 1 for a CONSORT flow diagram of study participation.

For the main study, mean age was 49 years, and most were female (71%) and had a white ethnicity (91%). Thirty-two participants (41%) were lower SEP (i.e. having some college or associate degree or less) and 46 (59%) were higher SEP (i.e. having a bachelor’s degree or higher). The majority of participants had overweight or obesity (60%). Participants lost to follow-up were broadly similar to completers. In the comparison sub-study participants were similar in demographic profile as in the main study. See Table 1.

**Table 1:** Baseline characteristics of participants in the main study (*n* = 78), those lost to follow up (*n* = 14) and those in the comparison study (*n* = 22)

	**Main study** **completers (*n* = 78)**	**Main study** **lost to follow-up (*n* = 14)**	**Comparison group (*n* = 22)**
Age, mean (SD), years	48.7 (16.1)	44.2 (16.2)	54.0 (19.6)
Sex, n (%)			
- *Female*	55 (70.5%)	7 (50.0%)	18 (81.8%)
* - Male*	23 (29.5%)	7 (50.0%)	4 (18.2%)
Ethnicity, n (%)			
* - White*	71 (91.0%)	11 (78.6%)	19 (86.4%)
* - Black/African/Caribbean/Black British*	0 (0.0%)	0 (0.0%)	0 (0.0%)
* - Asian/Asian British*	7 (9.0%)	3 (21.4%)	3 (13.6%)
* - Mixed/Multiple ethnic groups*	0 (0.0%)	0 (0.0%)	0 (0.0%)
Education, n (%)			
* - Less than high school*	2 (2.6%)	0 (0.0%)	0 (0.0%)
* - High school completion*	16 (20.5%)	1 (7.1%)	1 (4.6%)
* - Some college or associate degree*	14 (18.0%)	4 (28.6%)	7 (31.8%)
* - Bachelor’s degree*	24 (30.8%)	2 (14.3%)	8 (36.4%)
* - Master’s degree*	18 (23.1%)	6 (42.9%)	6 (27.3%)
* - Doctoral degree*	4 (5.1%)	1 (7.1%)	0 (0.0%)
Equivalised net monthly household income			
* - *Mean (SD), £	2216 (1667)	2712 (1612)	1839 (1283)
* - *Missing	7 (8.9%)	0 (0.0%)	6 (27.3%)
Employment status, n (%)			
* - Full-time*	31 (39.7%)	5 (35.7%)	4 (18.2%)
* - Part-time*	16 (20.5%)	4 (28.6%)	3 (13.6%)
* - Student*	8 (10.3%)	1 (7.1%)	4 (18.2%)
* - Retired*	20 (25.6%)	2 (14.3%)	9 (40.9%
* - Temporary/permanently sick or disabled*	0 (0.0%)	0 (0.0%)	0 (0.0%)
* - Looking after home/family*	0 (0.0%)	0 (0.0%)	0 (0.0%)
* - Other*	3 (3.9%)	2 (14.3%)	2 (9.1%)
Area-level deprivation (IMD), n (%)			
* - Quintile 1 (most deprived)*	30 (40.5%)	7 (50.0%)	4 (18.2%)
* - Quintile 2*	10 (13.5%)	3 (21.4%)	2 (9.1%)
* - Quintile 3*	16 (21.6%)	2 (14.3%)	2 (9.1%)
* - Quintile 4*	11 (14.9%)	0 (0.0%)	10 (45.5%)
* - Quintile 5 (least deprived)*	7 (9.5%)	2 (14.3%)	3 (13.6%)
* - Missing*	4 (5.1%)	0 (0.0%)	1 (4.5%)
Subjective socioeconomic status (0–10), mean (SD)	6.1 (1.2)	6.2 (1.8)	6.1 (1.3)
BMI, mean (SD)	27.8 (6.7)	27.6 (4.3)	26.4 (5.1)
Underweight, n (%)	1 (1.3%)	0 (0.0%)	1 (4.6%)
Normal weight, n (%)	30 (38.5%)	5 (35.7%)	7 (31.8%)
Overweight, n (%)	29 (37.2%)	5 (35.7%)	10 (45.5%)
Obesity, n (%)	18 (23.1%)	4 (28.6%)	4 (18.2%)

*BMI* body mass index, *IMD* index of multiple deprivation, *SD* standard deviation

Baseline characteristics by SEP are shown in the supplementary materials, Table S1. Participants with higher SEP were generally younger, more likely to be a student and were less likely to have obesity compared with lower SEP.)

### The effect of the pricing intervention on nutritional intake and money spent

**Fig. 2:**
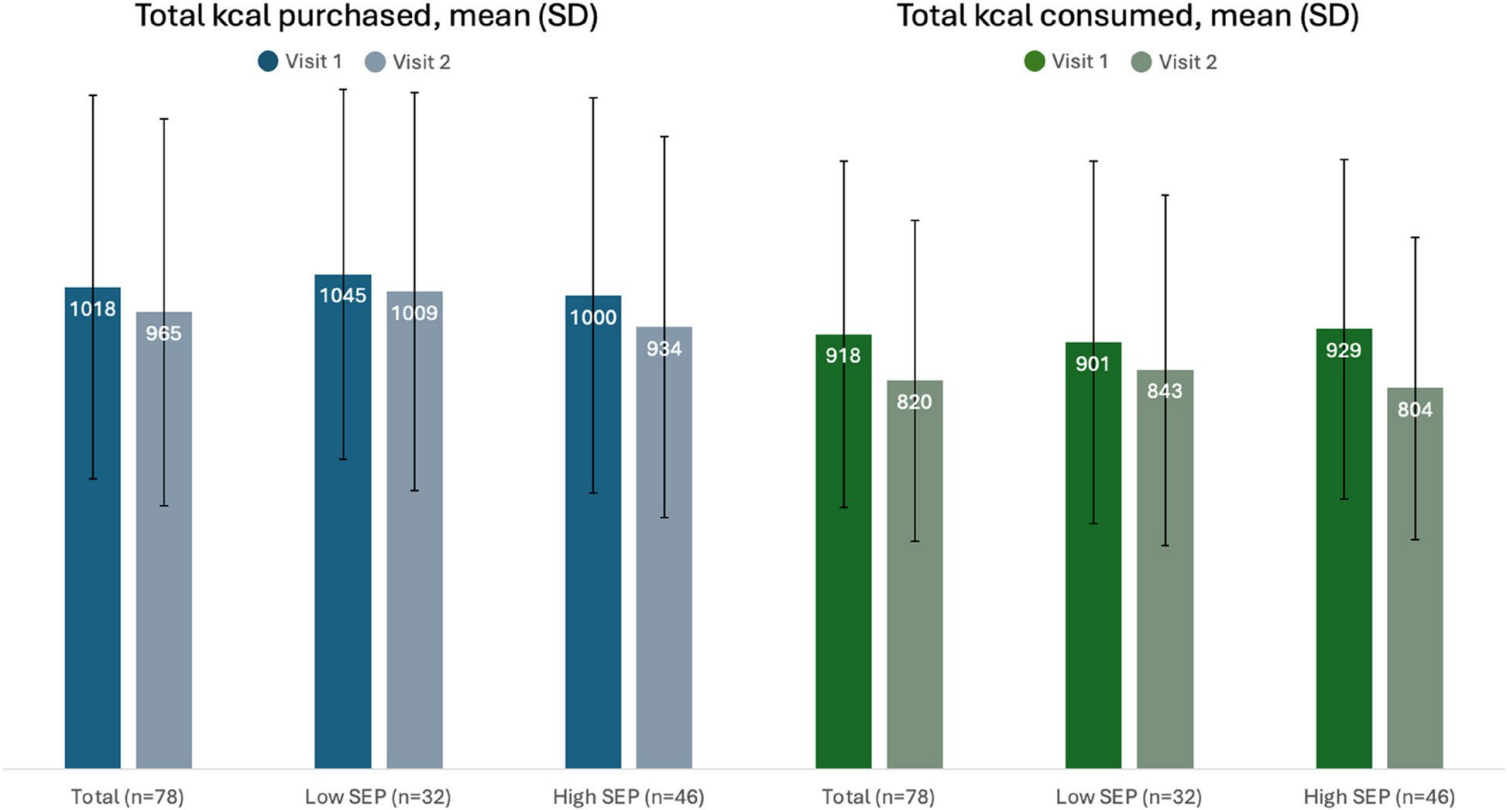
Total kcal purchased and total kcal consumed (means and standard deviations) at visit 1 and 2 for the total sample of the main study (not including those who were lost to follow up) and stratified by socioeconomic group. SEP, socioeconomic position; kcal, kilocalories

Descriptive statistics of primary and secondary outcome variables at visit 1 and 2 are shown in Fig. 2 and Table 2. Mean kcal purchased and consumed reduced at visit 2 (i.e. where the pricing intervention was implemented) compared to visit 1 (i.e. where the pricing intervention was not implemented) in the total sample, lower SEP group and higher SEP group (Fig. 2). However, as shown in Table 3, the effect was only statistically significant for kcal consumption: −98.0 ((95% CI −181.9, −14.2), *p* = 0.02). Although in the same direction of a decrease in kcals purchased after introduction of the pricing intervention, there was no evidence of a statistically significant effect on total kcal purchased (Table 3).

**Table 2:** Descriptive statistics of secondary outcome variables of the total sample of the main study (not including those who were lost to follow up) and stratified by socioeconomic groups

	**Total (*n* = 78)**		**Low SEP (*n* = 32)**^a^		**High SEP (*n* = 46)**^a^	
	**V1, mean (SD)**	**V2, mean (SD)**	**V1, mean (SD)**	**V2, mean (SD)**	**V1, mean (SD)**	**V2, mean (SD)**
*Secondary outcome variables*						
Total fat consumed (grams)	45.5 (26.5)	37.3 (22.0)	46.0 (30.4)	37.8 (23.7)	45.1 (23.8)	37.0 (21.0)
Total saturated fat consumed (grams)	20.2 (17.0)	15.5 (13.1)	22.2 (18.8)	15.7 (13.8)	18.8 (15.6)	15.4 (12.7)
Total salt consumed (grams)	2.1 (1.2)	1.9 (1.1)	2.0 (1.1)	2.1 (1.2)	2.1 (1.2)	1.8 (1.1)
Total sugar consumed (grams)	24.4 (19.9)	25.9 (25.6)	24.7 (19.3)	26.2 (21.5)	24.2 (20.4)	25.7 (28.3)
Kcal intake after visit	535.1 (421.0)	511.3 (445.3)	532.4 (396.9)	419.0 (361.8)	536.9 (441.3)	579.3 (491.6)
* - *missing	4 (5.1%)	12 (15.4%)	2 (6.3%)	4 (12.5%)	2 (4.3%)	8 (17.4%)
Total money spent (£)	21.4 (5.2)	19.6 (5.5)	21.8 (4.9)	19.3 (5.8)	21.2 (5.4)	19.9 (5.4)

*SD* standard deviation, *SEP* socioeconomic position, *kcal* kilocalories, *V1* visit 1, *V2* visit 2

^a^Low SEP is defined as having “some college or associate degree” or less, and high SEP is defined as having a “bachelor’s degree” or higher

**Table 3:** The effect of a 30% price decrease of lower kcal dishes on primary and secondary outcome variables of the total imputed sample of the main study and stratified by socioeconomic groups

	**Total (*n* = 92)**^a^		**Low SEP (*n* = 37)**^b^	**High SEP (*n* = 55)**^b^
	**Regression coefficient (95% CI)**	***p*-value**	**Regression coefficient (95% CI)**	**Regression coefficient (95% CI)**
*Primary outcome variables*				
Total kcal purchased	−47.8 (−146.5, 50.9)	0.34	−41.7 (−192.4, 108.9)	−51.9 (−181.4, 77.6)
Total kcal consumed	**−98.0 (−181.9, −14.2)**	**0.02**‡	−74.7 (−212.8, 63.5)	−113.8 (−221.3, −6.2)
*Secondary outcome variables*				
Total fat consumed (grams)	**−8.5 (−14.4, −2.6)**	**0.005**‡	−8.7 (−18.7, 1.3)	−8.3 (−15.6, −1.0)
Total saturated fat consumed (grams)	**−4.8 (−8.3, −1.2)**	**0.009**‡	−6.6 (−12.7, −0.5)	−3.5 (−7.9, 0.9)
Total salt consumed (grams)	−0.1 (−0.5, 0.2)	0.38	−0.0 (−0.6, 0.5)	−0.2 (−0.6, 0.2)
Total sugar consumed (grams)	1.3 (−4.2, 6.7)	0.65	1.8 (−7.1, 10.6)	0.9 (−6.2, 8.0)
Kcal intake after visit	−24.1 (−137.1, 88.8)	0.67	−126.8 (−259.1, 5.6)	51.2 (−103.3, 205.8)
Total money spent (£)	**−1.7 (−2.8, −0.6)**	**<0.001**‡	−2.4 (−4.3, −0.5)	−1.3 (−2.6, −0.0)

*CI* confidence interval, *kcal* kilocalories, *SEP* socioeconomic position

^‡^Regression coefficients in bold are statistically significant. For the primary outcome variables, statistical significance was set at *p* < 0.05 and for the secondary outcome variables, statistical significance was set at *p* < 0.01 to account for multiple testing

^a^14 participants who were lost to follow-up are imputed

^b^Low SEP is defined as having “some college or associate degree” or less, and high SEP is defined as having a “bachelor’s degree” or higher

As reported in Table 3, total fat and saturated fat consumed reduced on average by −8.5 g ((95% CI −14.4, −2.6), *p* = 0.005) and −4.8 g ((95% CI −8.3, −1.2), *p* = 0.009) at visit 2 compared to visit 1. There was no statistically significant difference between visits for total salt and sugar consumed or total kcal intake after the restaurant visit. Mean total money spent was statistically significantly lower in visit 2 compared to visit 1. Results appeared largely comparable (for measures of effect) between participants from lower vs. higher SEP for all outcomes.

### Comparison sub-study

As shown in Table 4, data from the comparison sub-study (*n* = 22) also indicated a directional reduction in kcal purchased and consumed and fat and saturated fat consumed from visit 1 to visit 2 in the absence of the price reduction intervention. As expected, there was no clear numerical difference in total money spent between the two visits. For descriptive purposes we computed effect estimates for outcomes; confidence intervals were relatively wide and should therefore be interpreted with caution.

**Table 4:** Descriptive statistics and effect sizes for comparator group (*n* = 22)

	**V1, mean (SD)**	**V2, mean (SD)**	**Regression coefficient (95% CI)**
*Primary outcome variables*			
Total kcal purchased	921.0 (353.6)	745.0 (308.5)	−176.1 (−349.8, −2.4)
Total kcal consumed	783.3 (350.0)	665.5 (268.4)	−117.8 (−258.3, 22.8)
*Secondary outcome variables*			
Total fat consumed (grams)	40.0 (21.3)	29.0 (15.3)	−11.0 (−20.3, −1.7)
Total saturated fat consumed (grams)	17.4 (13.8)	12.3 (9.3)	−5.2 (−11.4, 1.0)
Total salt consumed (grams)	2.2 (2.8)	1.3 (0.9)	−0.9 (−2.0, 0.2)
Total sugar consumed (grams)	22.7 (17.3)	23.3 (18.8)	0.6 (−4.7, 5.9)
Kcal intake after visit	537.5 (342.7)	546.5 (284.5)	9.1 (−179.5, 197.6)
- Missing	1 (4.5%)	2 (9.1%)	
Total money spent	20.9 (6.2)	21.5 (4.9)	0.6 (−1.1, 2.2)

*CI* confidence interval, *Kcal* kilocalories, *SEP* socioeconomic position, *SD* standard deviation

### Sensitivity analyses

Results of completers (*n* = 78) and those who completed the study and did not guess the study aim (*n* = 76) from the main study are show in the supplementary materials, Tables S2 and S3. Results were similar to the main analysis.

## Discussion

### Summary of results

The aims of this pilot study were to collect data on the potential effect of a price reduction intervention based on energy content in the OOHFS on kcal purchasing and kcal consumption to determine whether future larger studies are warranted and to inform their design. In the main study sample, we found that the kcal purchased and consumed tended to decrease from visit 1 (normal prices) to visit 2 (price reduction intervention) and this reduction was statistically significant for total kcal consumed. On average, participants consumed 98 kcal less when lower kcal dishes were discounted by 30% compared to when they visited the restaurant with standard prices in place. The reason why kcal purchased was not statistically significant may be because we did not have enough power to show a smaller effect. Results of the secondary outcomes indicated that the introduction of the pricing intervention was associated with reduced total and saturated fat intake, but not significantly so with sugar, salt or later energy intake. These outcomes were important to study to understand whether a kcal based intervention approach would be likely to not only decrease energy intake, but also intake of nutrients of concern. There was no notable difference between participants with higher vs. lower SEP, although we were not statistically powered to formally test sub-group differences by SEP. Importantly, in a small sub-study comparison group of participants who followed the same procedure but did not receive a price reduction intervention at visit 2, there was also a directional decrease in intake of kcal, fat and saturated fat. It is unclear why similar reductions in kcal ordered and consumed were observed in the absence of intervention. One possible reason for this is that participants may have been felt more aware of their dietary choices at visit 2 having completed measures previously and therefore selected healthier menu options [59] or it may be that consumers perceived the meal at visit 1 as a novel “treat” and their return visit (visit 2) less so Collectively, these findings suggest that although a larger scale trial testing the effects of healthier food price reductions in the OOHFS may be warranted and the results of the present study can inform likely effect sizes of interest, the inclusion of a control group (i.e. RCT, as opposed to pre-post design) will be particularly important to enable accurate estimation of the effect of a price reduction intervention.

Previous systematic reviews and meta-analyses have shown the effectiveness of price reductions on healthier food purchasing behaviour and consumption [28, 30, 31, 60, 61]; however, most of the included studies were situated in supermarkets and focussed on fruit and vegetable purchases. The most recent meta-analysis [28] included six studies that focused on “healthful foods” in canteens and supermarkets (e.g. salad bars, lower energy density, low in fat) and found that a standardised 20% price reduction increased healthy food purchase or consumption by 12%. Importantly, the present study suggests that a pricing intervention is potentially also effective to encourage healthier eating in a restaurant setting.

An aim of this study was to estimate potential effect sizes of interest to inform sample size calculation for future studies, because there is lack of existing research examining the impact of price reduction interventions on energy intake in the OOHFS. There is some debate on whether to use effect sizes from pilot studies to inform power calculations for larger studies, as the small sample sizes typically used in pilot studies can provide inaccurate effect size estimations [62, 63]. However, in this pilot study we were reasonably powered to detect effect sizes of pricing interventions reported in OOHFS settings previously and the final sample size of the main study group in the present study was relatively large (*n* = 92). We therefore propose that the present study can provide useful estimates for future trials. We did not have a sufficiently large enough sample size to estimate intervention effect differences by SEP with confidence for a future trial and this is a limitation of the present work. Nonetheless, from the limited number of higher vs. lower SEP participants recruited, there was not an obviously large difference in intervention effects on outcomes of interest. This suggests that if pricing reduction interventions in the OOHFS do differ by SEP, differences may be relatively small in statistical size and future studies should account for this.

### Strengths and limitations

To our knowledge, this is the first real-world pilot study investigating a price reduction intervention on lower kcal menu items to encourage healthy eating in a full-service restaurant in England and examine impacts on immediate energy and nutrient consumption, as well as potential compensatory effects for later energy intake. Studying population-based nutrition interventions in the real-world is important for external validity and to understand their effectiveness in a naturalistic environment, as a significant number of studies have examined intervention effects in hypothetical or laboratory settings [33, 37]. However, as the study was conducted at a single restaurant in Liverpool, with a predominantly white and female sample, further larger studies should determine whether the results are generalisable to broader populations and other geographical areas. A further strength is that the study design enabled estimation of individual participant energy consumption (rather than focussing on purchasing only, as is the case in many studies [64–66]) by developing a protocol for measurement of food waste (using photography) and group sharing of meals. Nonetheless, food photography measures are still likely to be prone to some error [67], but in the present study verification by a second researcher was used to address this. The methods adopted had relatively low loss to follow-up (14/114, 12%) and missing data (5% for all variables, except for income data which was > 10% for main study participants and self-reported later kcal intake, which 12 participants did not complete for visit 2 (15.4%)), which indicate that the adopted methodology could be feasible to use in larger trials. The main reason for loss to follow-up was that we were not able to book participants in for their second visit due to the restaurant closing down; attrition rates were similar for high and low SEP participants and baseline characteristics were largely comparable to those completing the study. We therefore believe attrition was random and multiple imputation provides reliable estimates [68]. Further strengths include measurement of participant awareness of study aims. Very few participants were aware of study aims (1.8%) and therefore demand characteristics are unlikely to explain findings. The inclusion of participants diverse in SEP is a further strength.

A limitation of the study is that the original design of the study was not planned to be a pilot study, and we were forced to change the study design early in data collection due to unforeseen circumstances. This resulted in us changing planned analyses, but the present study does provide useful information that can inform future research. The results indicate that it would be valuable to repeat the intervention on a larger scale, include a control condition and understand interaction effects by SEP. Nutritional information for macronutrients was estimated from ingredient lists provided by the restaurant and objective verification would be preferable in future. Moreover, as is common in the OOHFS, although the restaurant used serving standardisation methods, meal size and composition may still have varied somewhat across study days due to variations in serving sizes by different restaurant staff. These variations are, however, likely to be relatively small and presumed to be random in either direction (larger vs smaller). As is standard, we reimbursed participants for their time. Given that this study examined the effect of pricing on food purchased and consumed, it is feasible that the reimbursement provided could have altered purchasing behaviour and resulted in some participants behaving differently to how they would typically (e.g., spending more due to awareness of reimbursement), which may have resulted in the pricing intervention having a less pronounced effect on behaviour. Future research would benefit from addressing this limitation. In the comparative sub-study participants received menus without price reductions at both visits, and the pattern of results indicated that there may have been pre-post change irrespective of intervention delivery, as kcal purchased and consumed tended to be lower at visit 2 than at visit 1. Due to the small sample size of the comparison study, results should however be interpreted with caution, but nonetheless indicate that future trials will benefit from adopting a control condition with the full study design (i.e., RCT design) as a pre-post design with a control condition will be prone to potential order effects. Lastly, the 30% price decrease for healthier menu items might be challenging for businesses to implement without supporting subsides. This is a limitation of the current study and further research examining smaller price increments, which might be more scalable, sustainable and cost-effective, in real-world settings may now be valuable.

### Future research

Future larger studies are now needed to build on the results of this pilot study. Although we worked collaboratively with the restaurant in the present study, one recommendation is that interventions are co-developed with restaurant owners and customers, as suggested by the UK Medical Research Council (MRC) for the design of complex interventions [69]. Previous research has shown that the process of co-development increases retailer satisfaction as well as may provide opportunities for creative solutions to arise by those directly involved in the retail setting [70]. In the present study there was a significant reduction in total money spent by customers during the restaurant visit in which the pricing reduction intervention was implemented, which would presumably negatively impact the revenues of restaurants. It has been found that maintenance of profits is an important consideration for restaurant owners for nutrition interventions to be feasible and maintainable [71]. Therefore, future research may benefit from understanding how to promote healthier eating in restaurant settings without negatively affecting restaurant revenue.

Previous studies suggested that combining pricing with an “awareness campaign” (e.g., advertisement of the price reductions and education on why dishes are reduced in price) may be more effective than a pricing intervention alone [30]. In a future study it would therefore be valuable to test whether combining a pricing study with such an awareness campaign would lead to greater effects.

### Implications and conclusions

To improve population health, nutrition policies should be enacted across a wide range of food environments. Fiscal policies have already proven to be effective in supermarkets [28, 30, 31, 60, 61]; however, there is limited evidence on the effect of such interventions in the OOHFS, such as restaurants. The frequency people eat out of the home in the UK has increased in recent years [13]. As eating out of the home is associated with unhealthy food choices [72], it is an important setting for nutrition interventions. Overall, this pilot study found that a lower energy menu item price reduction intervention is potentially an effective strategy to encourage healthier eating in a restaurant setting. Future larger studies are now needed to formally evaluate effectiveness and whether this intervention approach is equitable across different socioeconomic groups.

## Supplementary Information

Supplementary Material 1.

## Data Availability

Data is available on the Open Science Framework (OSF), https://osf.io/7vh3p/.

## Abbreviations

BMI: Body mass index
IMD: Index of multiple deprivation
Kcal: Kilocalories
OOHFS: Out-of-home food sector
SD: Standard deviation
SEP: Socioeconomic position
