# Supplementary material for: The effect of healthier menu item price reductions in the out-of-home food sector on energy purchased and consumed: a restaurant-based pilot experiment
Source: BMC Public Health. 2025 May 22;25:1893. doi: 10.1186/s12889-025-21992-1 (PMC12096655; doi:10.1186/s12889-025-21992-1)
Supplement: Supplementary file 1 — Supplementary Material 1. [file 12889_2025_21992_MOESM1_ESM.docx]

# Supplementary materials


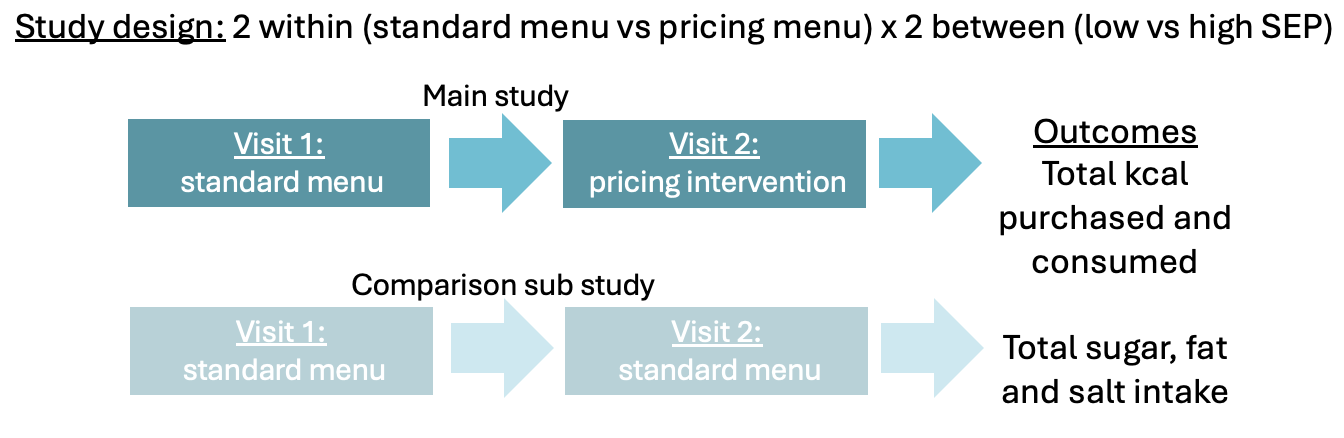


Figure S1: Study design of the main study and the comparison sub study


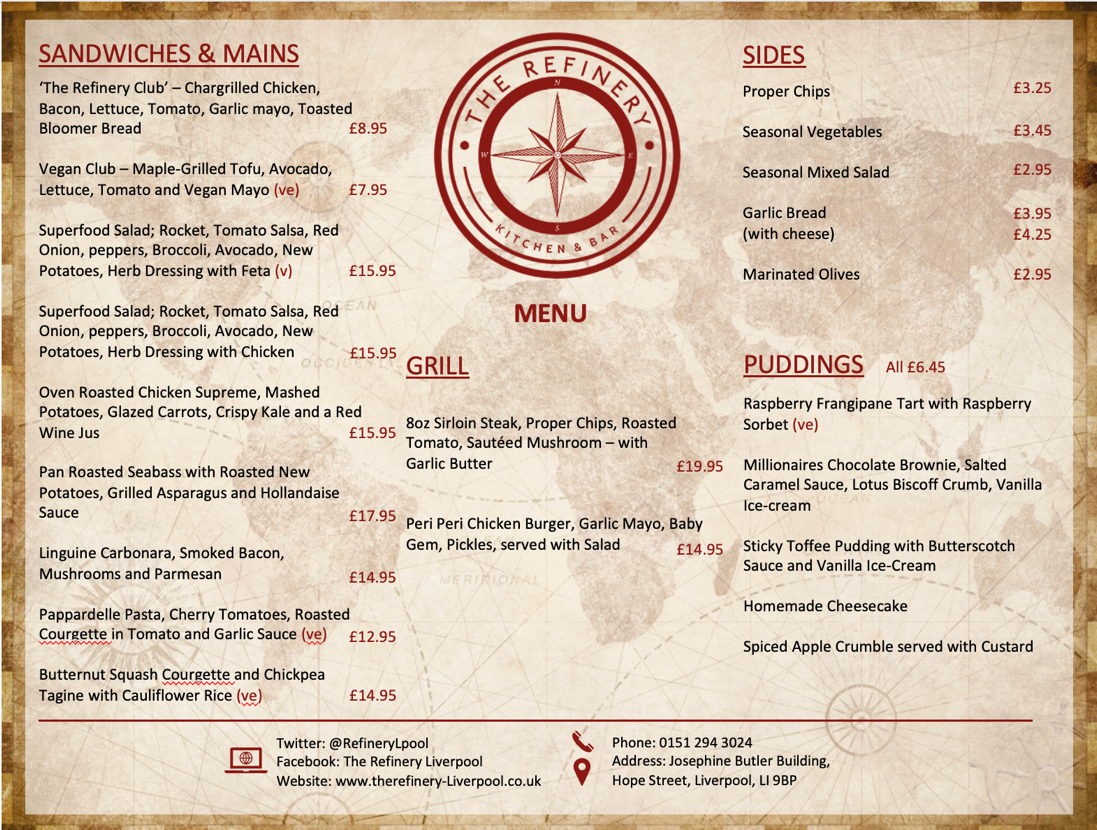


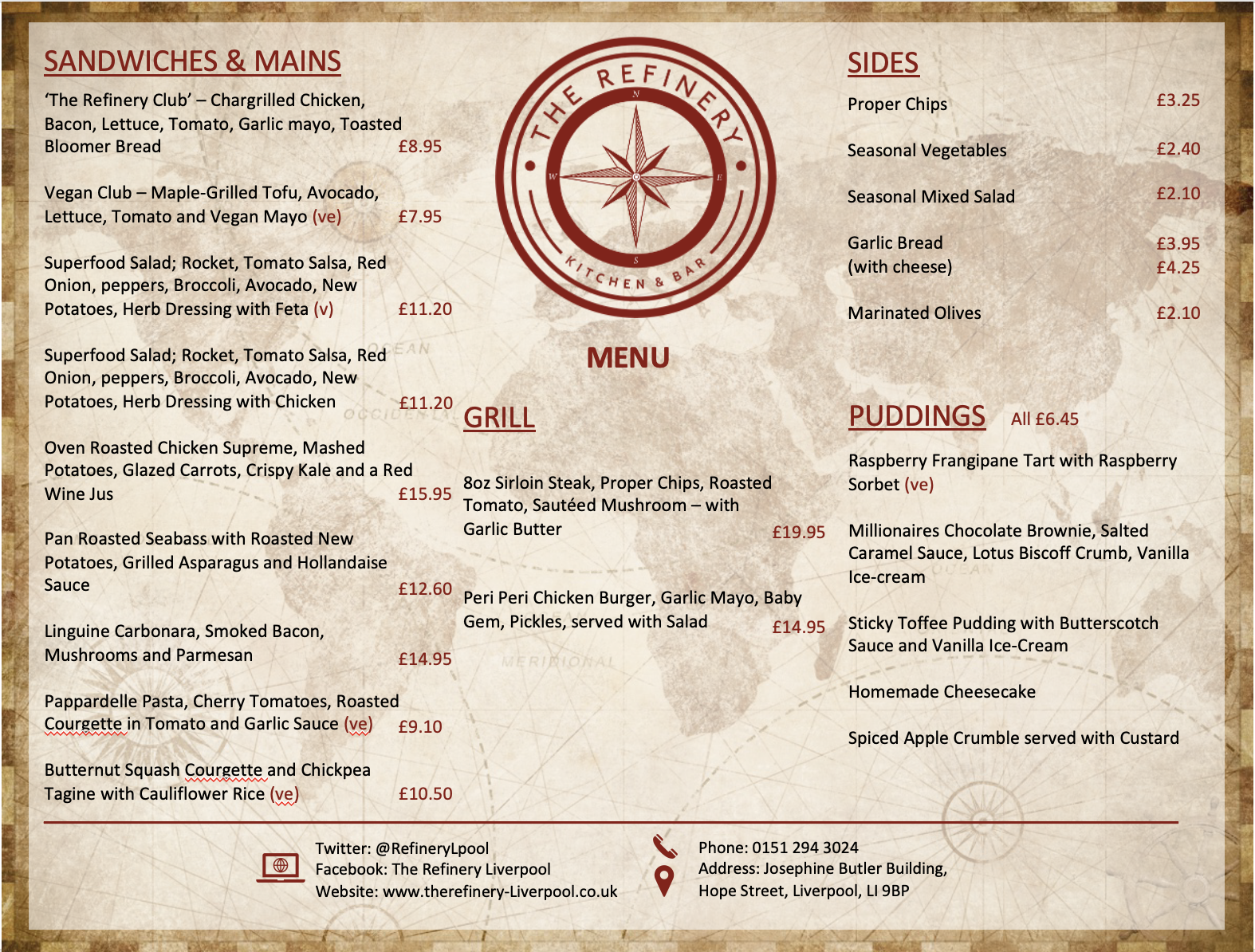

Figure S2: Menu A with standard prices (top) and with 30% discount on lower kcal products (bottom)


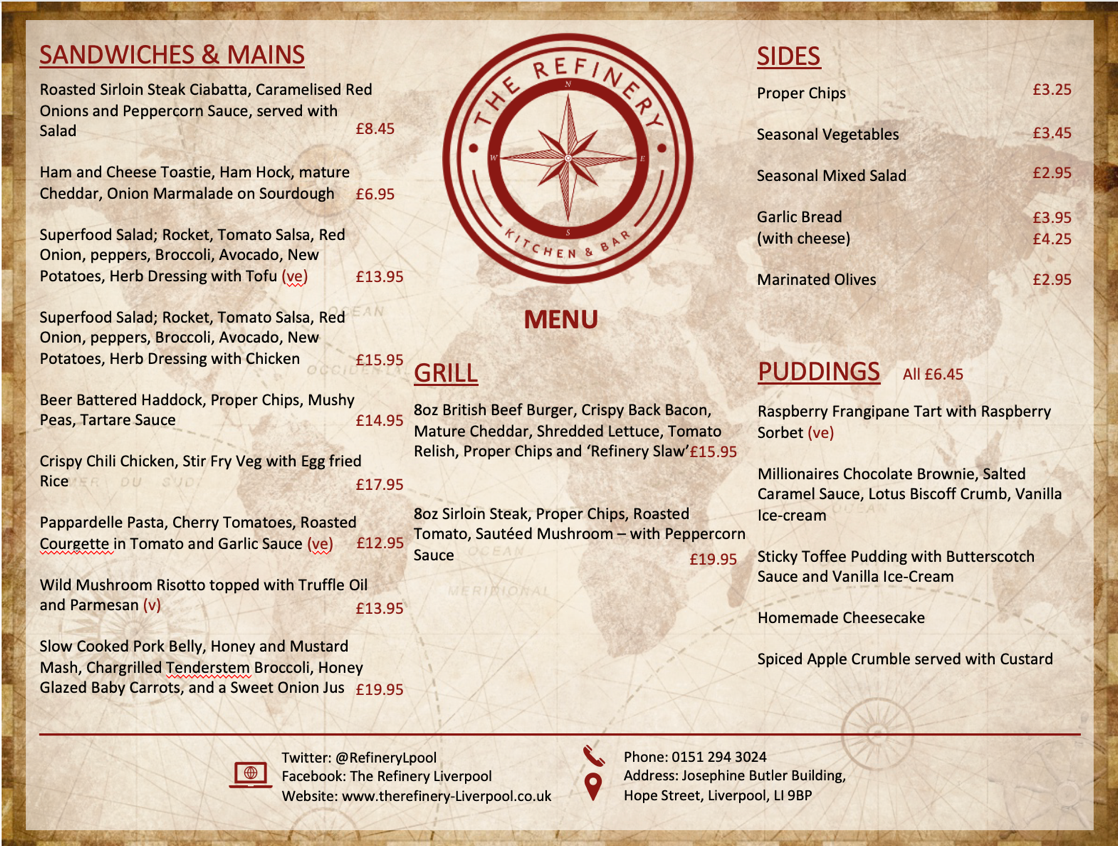


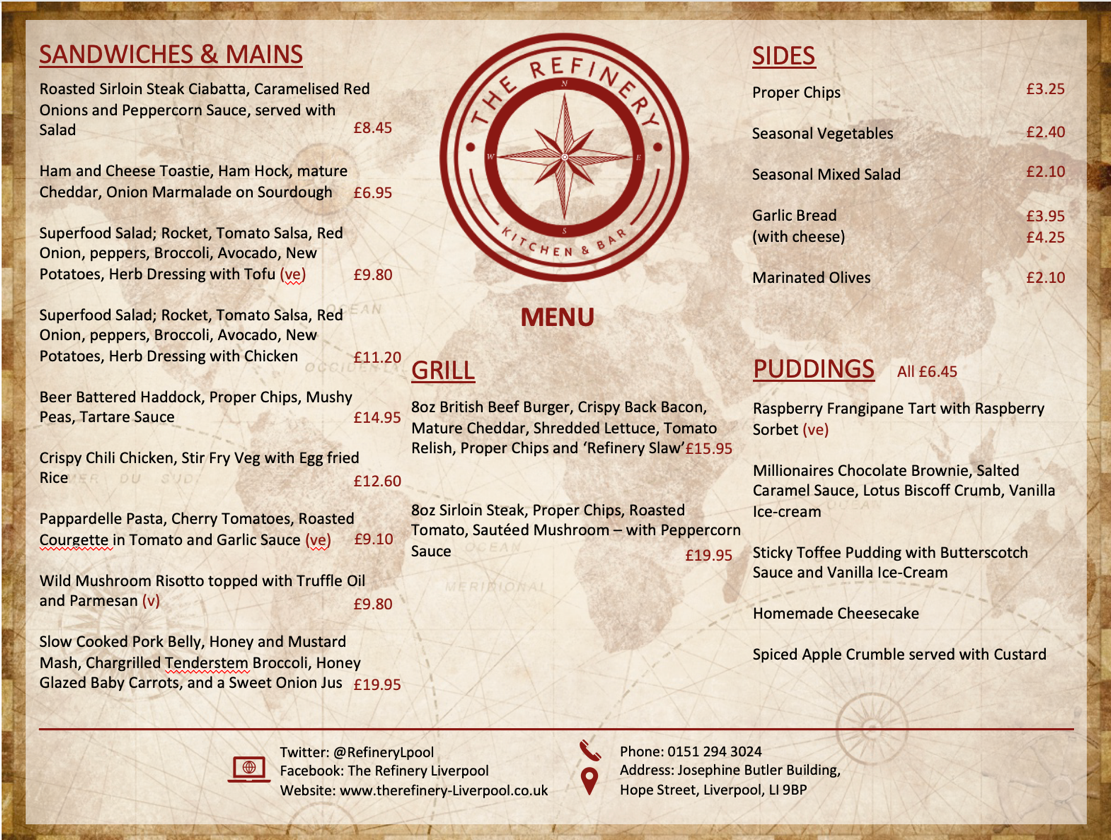


Figure S3: Menu B with standard prices (top) and with 30% discount on lower kcal products (bottom)

| Box S1: Deviations to analysis plan of original protocol due to loss of power |
| --- |
| - Reduced sample size - No testing of interaction effects by socioeconomic position (and by sex, body mass index, ethnic group in sensitivity analyses) - No mediation analyses to understand whether potential interaction effect by socioeconomic position is due to differences in food motives between high and low socioeconomic position (as no power for interaction analyses) - No Bayesian analyses to understand more compelling evidence of no interaction effect by SEP (as no power for interaction analyses) |

Table S1: Baseline characteristics by socioeconomic position

|  | **Low SEP (n=32)*** | **High SEP (n=46)*** |
| --- | --- | --- |
| Age, mean (SD), years | 59.5 (7.9) | 41.2 (16.1) |
| Sex, n (%) | | |
| - *Female* | 25 (78.1%) | 30 (65.2%) |
| - *Male* | 7 (21.9%) | 16 (34.8%) |
| Ethnicity, n (%) | | |
| - *White* | 32 (100%) | 39 (84.8%) |
| - *Black/African/Caribbean/Black British* | 0 (0.0%) | 0 (0.0%) |
| - *Asian/Asian British* | 0 (0.0%) | 7 (15.2%) |
| - *Mixed/Multiple ethnic groups* | 0 (0.0%) | 0 (0.0%) |
| Equivalised net household income | | |
| - Mean (SD), £ | 2470 (1600) | 2040(1708) |
| - Missing | 3 (9.4%) | 4 (9.5%) |
| Employment status, n (%) | | |
| - *Full-time* | 12 (37.5%) | 19 (41.3%) |
| - *Part-time* | 6 (18.8%) | 10 (21.7%) |
| - *Student* | 0 (0.0%) | 8 (17.4%) |
| - *Retired* | 14 (43.8%) | 6 (13.0%) |
| - *Temporary/permanently sick or disabled* | 0 (0.0%) | 0 (0.0%) |
| - *Looking after home/family* | 0 (0.0%) | 0 (0.0%) |
| - *Other* | 0 (0.0%) | 3 (6.5%) |
| Area-level deprivation (IMD), n (%) | | |
| - *Quintile 1 (most deprived)* | 13 (41.9%) | 17 (39.5%) |
| - *Quintile 2* | 2 (6.5%) | 8 (18.6%) |
| - *Quintile 3* | 7 (22.6%) | 9 (20.9%) |
| - *Quintile 4* | 5 (16.1%) | 6 (14.0%) |
| - *Quintile 5 (least deprived)* | 4 (12.9%) | 3 (7.0%) |
| - *Missing* | 1 (3.1%) | 3 (6.5%) |
| Subjective socioeconomic status (0–10), mean (SD) | 5.7 (1.3) | 6.3 (1.1) |
| BMI, mean (SD) | 29.0 (5.8) | 27.0 (7.2) |
| Underweight, n (%) | 1 (3.1%) | 0 (0.0%) |
| Normal weight, n (%) | 8 (25.0%) | 22 (47.8%) |
| Overweight, n (%) | 11 (34.4%) | 18 (39.1%) |
| Obesity, n (%) | 12 (37.5%) | 6 (13.0%) |

BMI, body mass index; IMD, index of multiple deprivation; SD, standard deviation. *Low SEP is defined as having “some college or associate degree” or less, and high SEP is defined as having a “bachelor’s degree” or higher.

Table S2: The effect of a 30% price decrease of lower kcal dishes on primary and secondary outcome variables of those who completed the study (n=78) and stratified by socioeconomic groups using linear mixed models

|  | **Total completers (n=78)** | | **Low SEP (n=32)*** | **High SEP (n=46)*** |
| --- | --- | --- | --- | --- |
|  | **Regression coefficient (95% CI)** | **p-value** | **Regression coefficient (95% CI)** | **Regression coefficient (95% CI)** |
| *Primary outcome variables* | | | | |
| Total kcal purchased | -53.5 (-155.4, 48.4) | 0.30 | -36.0 (-190.8, 118.7) | -65.6 (-200.6, 69.5) |
| Total kcal consumed | **-97.4 (-182.3, -12.6)** | **0.03†** | -58.0 (-198.5, 82.4) | -124.9 (-229.7, -20.1) |
| *Secondary outcome variables* | | | | |
| Total fat consumed (grams) | **-8.2 (-14.1, -2.2)** | **0.01†** | -8.2 (-18.6, 2.1) | -8.2 (-15.3, -1.1) |
| Total saturated fat consumed (grams) | **-4.7 (-8.3, -1.0)** | **0.01†** | -6.4 (-12.7, -0.1) | -3.4 (-7.8, 0.9) |
| Total salt consumed (grams) | -0.2 (-0.5, 0.2) | 0.34 | -0.0 (-0.5, 0.6) | -0.3 (-0.7, 0.1) |
| Total sugar consumed (grams) | 1.5 (-3.9, 6.9) | 0.59 | 1.4 (-7.4, 10.2) | 1.5 (-5.2, 8.3) |
| Kcal intake after visit | -5.3 (-113.5, 102.9) | 0.92 | -122.3 (-241.3, -3.2) | 73.4 (-85.3, 232.0) |
| Total money spent | **-1.8 (-2.9, -0.7)** | **0.00†** | -2.5 (-4.5, -0.5) | -1.3 (-2.6, -0.0) |

CI, confidence interval; kcal, kilocalories; SEP, socioeconomic position. *Low SEP is defined as having “some college or associate degree” or less, and high SEP is defined as having a “bachelor’s degree” or higher. †Regression coefficients in bold are statistically significant. For the primary outcome variables, statistical significance was set at p<0.05 and for the secondary outcome variables, statistical significance was set at p<0.01 to account for multiple testing.

Table S3: The effect of a 30% price decrease of lower kcal dishes on primary and secondary outcome variables of those who completed the study and who did not guess the study aim (n=76) and stratified by socioeconomic groups using linear mixed models

|  | **Total non-aim guessers (n=76)** | | **Low SEP (n=31)*** | **High SEP (n=45)*** |
| --- | --- | --- | --- | --- |
|  | **Regression coefficient (95% CI)** | **p-value** | **Regression coefficient (95% CI)** | **Regression coefficient (95% CI)** |
| *Primary outcome variables* | | | | |
| Total kcal purchased | -51.9 (-156.4, 52.7) | 0.33 | -33.7 (-193.3, 126.0) | -64.4 (-202.4, 73.7) |
| Total kcal consumed | **-95.0 (-181.9, -8.2)** | **0.03†** | -58.0 (-203.0, 87.0) | -120.5 (-227.3, -13.7) |
| *Secondary outcome variables* | | | | |
| Total fat consumed (grams) | -7.4 (-13.4, -1.4) | 0.02 | -7.1 (-17.6, 3.3) | -7.6 (-14.7, -0.4) |
| Total saturated fat consumed (grams) | -4.2 (-7.8, -0.5) | 0.03 | -5.7 (-12.0, 0.6) | -3.1 (-7.5, 1.3) |
| Total salt consumed (grams) | -0.2 (-0.5, 0.2) | 0.32 | -0.0 (-0.6, 0.5) | -0.3 (-0.7, 0.1) |
| Total sugar consumed (grams) | 0.3 (-4.8, 5.5) | 0.90 | 0.8 (-8.3, 9.8) | 0.1 (-6.1, 6.3) |
| Kcal intake after visit | 1.3 (-109.5, 112.2) | 0.98 | -112.2 (-234.2, 9.8) | 76.3 (-86.4, 238.9) |
| Total money spent | **-1.8 (-3.0, -0.7)** | **0.00†** | -2.3 (-4.4, -0.3) | -1.5 (-2.8, -0.2) |

CI, confidence interval; kcal, kilocalories; SEP, socioeconomic position. *Low SEP is defined as having “some college or associate degree” or less, and high SEP is defined as having a “bachelor’s degree” or higher. †Regression coefficients in bold are statistically significant. For the primary outcome variables, statistical significance was set at p<0.05 and for the secondary outcome variables, statistical significance was set at p<0.01 to account for multiple testing.
